# Ultrasound-guided attenuation parameter for hepatic steatosis quantification in psoriasis: a retrospective cohort study: Quantitatively evaluate of hepatic steatosis

**DOI:** 10.1097/MD.0000000000046896

**Published:** 2025-12-26

**Authors:** Xiaonan Wang, Yuting Jiao, Shengwei Zhou, Yaqi Fang, Huantong Duan, Zhi Yang

**Affiliations:** a Department of Ultrasound, Binzhou Medical University Hospital, Binzhou, Shandong Province, China; b Maternal and Child Health Hospital of Shigatse City, Shigatse City, Tibet Autonomous Region, China.

**Keywords:** hepatic steatosis, psoriasis, ultrasound, ultrasound-guided attenuation parameters

## Abstract

Ultrasound-guided attenuation parameter (UGAP), a validated hepatic steatosis quantification modality, demonstrates superior diagnostic accuracy in metabolic dysfunction-associated fatty liver disease (MAFLD). This study aimed to evaluate the occurrence and characteristics of hepatic steatosis in patients with psoriasis by UGAP. Psoriatic inpatients (March–October 2023) at Binzhou Medical University Hospital were enrolled, excluding those with secondary chronic liver disease etiologies. The control group, recruited from nonpsoriasis physical examiners, were matched for gender, age, and body mass index (BMI). The UGAP of all participants were measured. A total of 86 participants were included in this study, among whom 61 were patients with psoriasis and 25 were in the control group. The UGAP values of psoriasis patients were significantly higher than those of the nonpsoriasis group (0.65 vs 0.60, *P* = .01). Psoriasis severity index (PASI)-stratified analysis revealed severe psoriasis patients demonstrated prolonged disease duration and elevated UGAP versus mild-to-moderate counterparts (0.69 vs 0.63, *P* = .03). Spearman correlation analysis showed that in the psoriasis population, the correlation coefficient between the PASI scores and the UGAP values was 0.42 (*P* < .001). Age and UGAP values were significant predictors of a higher PASI score independent of gender, age at onset, duration of psoriasis and BMI. A bidirectional connection exists between hepatic steatosis and psoriasis progression. UGAP, as a promising quantitative assessment tool for hepatic steatosis, demonstrates significant correlations with psoriasis severity indices. This modality sensitively reflects the gradation of hepatic lipid deposition in psoriatic patients and exhibits predictive value for disease progression.

## 1. Introduction

The development of psoriasis as an inflammatory skin disease was closely correlated with metabolic disorders, and several studies have confirmed an isotropic link between psoriasis and nonalcoholic fatty liver disease (NAFLD).^[1–3]^ Currently, NAFLD has been reclassified as metabolic dysfunction-associated fatty liver disease (MAFLD) by international organizations, underscoring the critical role of metabolic dysfunction in the pathogenesis of this condition.^[4]^ Early MAFLD can be asymptomatic, and as the disease progresses, it can develop into cirrhosis or even liver cancer,^[5]^ thereby seriously affecting the prognosis and quality of life of patients. Meanwhile, as MAFLD progresses, the damaged liver cells can further release pro-inflammatory cytokines, driving low-grade inflammation and aggravating psoriasis, thereby seriously affecting the quality of life of patients. Therefore, evaluating the correlation between psoriasis and the occurrence of hepatic steatosis, and assessing hepatic steatosis early and correctly are of great significance to the treatment and prognosis of patients with psoriasis. Liver biopsy is the gold standard for quantitatively assessing the extent of hepatic steatosis, however, because of its invasiveness and poor reproducibility, it is not the first choice for clinical diagnosis.^[6]^ Conventional ultrasound has emerged as the preferred modality for hepatic steatosis in patients with psoriasis assessment due to its noninvasive and user-friendly characteristics.^[3,7,8]^ However, it heavily relies on subjective judgement of diagnosticians and exhibits limited sensitivity in diagnosing mild fatty liver disease. Ultrasound-guided attenuation parameter (UGAP), as a new technology for the noninvasive detection of hepatic steatosis, is able to assess the degree of hepatic steatosis in a quantitative manner,^[9]^ which compensates for the limitations of conventional ultrasound to a certain extent. This study aimed to apply the UGAP technique to explore the correlation between the occurrence of psoriasis and the degree of hepatic steatosis.

## 2. Materials and methods

### 2.1. Study design and patients

Patients with plaque psoriasis who were admitted to the Binzhou Medical University Hospital from March 1, 2023 to October 31, 2023 were retrospectively enrolled. All patients with psoriasis were seen by dermatologist for the first time and reported no prior use of related medications. Patients who had any clinical evidence of malignancy, cirrhosis or other secondary causes of chronic liver disease (i.e., alcohol abuse, viral hepatitis or current use of potentially hepato-toxic medications such as tamoxifen, methotrexate, tumor necrosis factor [TNF] antagonists, and amiodarone) were excluded. All patients were confirmed not to have autoimmune liver disease both at initial presentation and during follow-up after treatment. Participants had no history of metabolic diseases (including diabetes mellitus and metabolic syndrome [MetS]) prior to psoriasis onset. All data collected from the electronic medical records were de-identified. Alcohol abuse was defined as consumption of >30 g alcohol per day for men and >20 g alcohol per day for women.^[10]^ The control group was recruited from nonpsoriasis physical examiners who met the above exclusion criteria. This study was carried out in accordance with the recommendations of the Declaration of Helsinki. The protocol was approved by the Institutional Review Board (or Ethics Committee) of Binzhou Medical University Hospital (KYLL-095). The individual consent for this retrospective analysis was waived.

### 2.2. UGAP measurements

**Figure 1.**
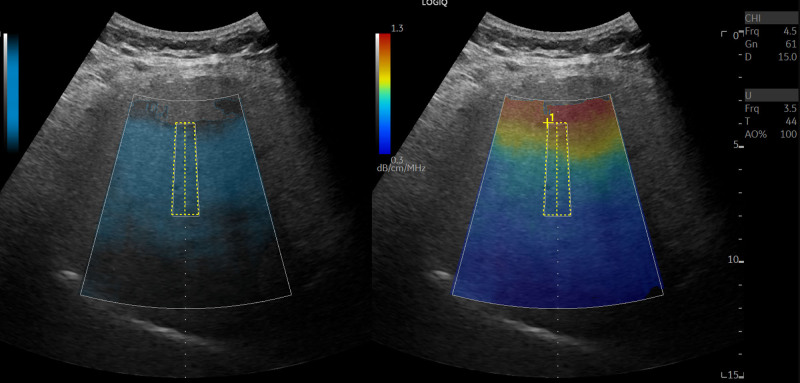
UGAP measurements. A large color-coded attenuation map was positioned for UGAP measurement in a homogeneous area of the liver, free of large vessels. UGAP = ultrasound-guided attenuation parameter.

LOGIQ E10s diagnostic ultrasound machine and liver sweep with C1-6-D probe with UGAP detection was used. Liver ultrasound scans were performed, and attenuation coefficients were obtained for all participants by a trained sonographer who was unaware of the participants’ condition and severity of psoriasis. The examination was performed after the subjects fasted for more than 4 hour in a supine position with the upper limbs fully elevated. Using the UGAP quality-map option, the region of interest was placed in a homogeneous area of the liver, free of large vessels. The best image was selected to obtain attenuation coefficient measurements (Fig. 1). Three measurements were made from the left lobe of the liver, the upper segment of the right lobe of the liver, and the lower segment of the right lobe of the liver, for a total of 9 measurements. The final UGAP measurement was defined as the median of the 9 measurements, expressed in dB/cm/MHz.^[11]^

### 2.3. Laboratory indicators

All subjects were fasted for liver function tests for serum alanine aminotransferase (ALT), aspartate aminotransferase (AST), alkaline phosphatase (ALP), and gamma-glutamyl-transferase (GGT), and fasting venous blood was collected from all psoriasis patients for serum total cholesterol (CHO), triglycerides (TG), high-density lipoprotein cholesterol (HDL-C), low-density lipoprotein cholesterol (LDL-C), small and dense LDL cholesterol, lipoprotein(a), and glucose.

### 2.4. Other information

The following general information of the patients was recorded: gender, age, height, weight, and body mass index (BMI). The severity of psoriasis was assessed using the psoriasis severity index (PASI). The scoring system stratifies the entire body into 4 regions (cephalic region, upper extremities, trunk, and lower extremities). Each region undergoes independent evaluation, with the composite score derived from the summation of all regional subscores, and those who met a PASI ≥ 10 were defined as patients with severe psoriasis; those with a PASI < 10 were defined as having mild-to-moderate psoriasis.^[12]^ All dermatologists remained blinded to UGAP values during their assessment of psoriasis severity using PASI.

### 2.5. Statistical analysis

Statistical analysis was performed using SPSS v.26.0. Quantitative data were tested for normality, and those conforming to normal distribution were expressed as the mean ± standard deviation. Non-normally distributed data were expressed as (M [P25, P75]). Comparisons between groups conforming to normal distribution were performed using the *t* test, and non-normally distributed data were tested using the rank-sum test. The Spearman rank correlation coefficient was used to determine the correlation between UGAP and psoriasis. Logistic regression was used to analyze the relationship between each variable and the severity of psoriasis. Independent influences were screened, and *P* < .05 were considered statistically significant.

## 3. Results

### 3.1. Characteristics of the psoriasis and control groups

From March 1, 2023 to October 31, 2023, 77 psoriasis patients referred to receive UGAP were screened. Finally, 61 patients with psoriasis were enrolled. The main exclusion reasons were alcohol abuse (N = 6), viral hepatitis (N = 6) or the clinical data were incomplete (N = 4). Meanwhile, 25 healthy controls were included. The mean age was 40.36 ± 12.66 years in the psoriasis group and 45.60 ± 14.64 years in the control group. The psoriasis group had 38 males (62.3%) and the control group had 12 males (48.0%). No statistically significant difference existed in age, gender, and BMI values between the psoriasis and control groups. UGAP values were significantly higher in the psoriasis group than in the control group (*P* < .05). GGT was significantly higher in the psoriasis group than in the control group (*P* < .05), but no significant differences were observed in serum AST, ALT, and ALP between the 2 groups (*P* > .05) (Table 1).

**Table 1:** Baseline characteristics of the study population.

	Psoriasis patients (n = 61)	Controls (n = 25)	*P*-value
Gender (male: female)	38:23	12:13	.12
Age (yr)	40.36 ± 12.66	45.60 ± 14.64	.10
Height (m)	1.69 ± 0.08	1.67 ± 0.07	.28
Weight (kg)	74.80 ± 15.24	69.36 ± 10.47	.06
BMI (kg/m^2^)	26.12 ± 4.99	24.77 ± 3.37	.15
UGAP (dB/cm/MHz)	0.65 ± 0.11	0.60 ± 0.07	.01
AST (U/L)	20.00 (16.4, 23.9)	21.10 (17.9, 23.9)	.43
ALT (U/L)	20.42 (13.2, 31.5)	16.20 (12.3, 22.5)	.11
ALP (U/L)	71.60 (57.9, 87.5)	83.70 (56.6, 98.0)	.24
GGT (U/L)	26.00 (17.8, 40.8)	19.60 (13.0, 28.1)	.03

ALT = alanine aminotransferase, AST = aspartate aminotransferase, BMI = body mass index, GGT = gamma-glutamyl-transferase, UGAP = ultrasound-guided attenuation parameter.

### 3.2. Comparison of clinical characteristics between the group of patients with mild-to-moderate psoriasis and the group with severe psoriasis

Thirty-six patients had mild-to-moderate psoriasis, among which 24 (66.7%) were male. Twenty-five patients had severe psoriasis, among which 14 (56.0%) were male. No statistically significant difference existed between the 2 groups in terms of height, weight, BMI, and age of onset. Patients with severe psoriasis were older than those with mild-moderate psoriasis, had a longer duration of disease than those with mild-moderate psoriasis, and had a significantly higher UGAP value than those with mild-moderate psoriasis (*P* < .05). However, no significant difference existed in the laboratory parameters between the 2 groups (Table 2).

**Table 2:** Clinical and biochemical characteristics of patients with psoriasis grouped by PASI.

	Mild–to-moderate psoriasis (n = 36)	Severe psoriasis (n = 25)	*P*-value
Gender (male: female)	24:12	14:11	.40
Age (yr)	37.11 ± 12.35	45.04 ± 11.81	.02
Psoriasis duration (yr)	11.56 ± 9.59	15.79 ± 8.99	.09
age of onset (yr)	25.56 ± 9.26	29.25 ± 8.92	.13
Height (m)	1.70 ± 0.08	1.68 ± 0.07	.39
Weight (kg)	72.90 ± 15.47	77.52 ± 14.77	.25
BMI (kg/m^2^)	25.28 ± 5.23	27.33 ± 4.44	.12
UGAP (dB/cm/MHz)	0.63 ± 0.11	0.69 ± 0.10	.03
AST (U/L)	19.95 (16.6, 24.2)	20.00 (16.1, 23.1)	.58
ALT (U/L)	20.20 (12.1, 33.2)	22.50 (14.5, 30.2)	.63
ALP (U/L)	71.40 (57.9, 86.1)	73.80 (57.9, 91.0)	.79
GGT (U/L)	25.10 (17.9, 40.9)	27.30 (17.5, 41.0)	.94
GLU (mmol/L)	4.94 (4.7, 5.4)	4.88 (4.5, 5.4)	.66
CHO (mmol/L)	4.88 (3.7, 5.3)	4.55 (3.8, 5.2)	.71
TG (mmol/L)	1.17 (0.8, 2.3)	1.24 (0.8, 2.5)	.59
HDL-C (mmol/L)	1.11 (0.9, 1.3)	1.16 (1.0, 1.3)	.18
LDL-C (mmol/L)	3.14 (2.5, 3.6)	3.03 (2.3, 3.5)	.52
sdLDL (mmol/L)	0.87 (0.6, 1.3)	1.06 (0.5, 1.4)	.81
LP (a) (mg/dL)	10.02 (5.6, 29.0)	19.84 (12.0, 28.2)	.05

ALP = alkaline phosphatase, ALT = alanine aminotransferase, AST = aspartate aminotransferase, BMI = body mass index, CHO = cholesterol, GGT = gamma-glutamyl-transferase, GLU = glucose, HDL-C = high-density lipoprotein cholesterol, LDL = cholesterol, LDL-C = low-density Lipoprotein cholesterol, LP (a) = lipoprotein (a), sdLDL = small and dense, TG = triglycerides, UGAP = ultrasound-guided attenuation parameter.

### 3.3. Factors influencing the severity of psoriasis

Table 3 summarizes the factors influencing PASI scores. We included factors with *P* < .05 in the one-way regression analysis in the multifactorial linear-regression analysis. In the multifactorial linear-regression analysis, age and UGAP value were independent predictors of psoriasis severity as measured by PASI score, whereas gender, BMI, age at onset, and duration of psoriasis were not.

**Table 3:** Univariate and multivariate analyses of factors influencing Psoriasis Area and Severity Index of psoriasis patients.

Risk factors	Univariate analyses		Multivariate analyses	
	OR	*P*-value	OR	*P*-value
Gender (male)	0.636	.40	NA	–
Age (yr)	1.055	.02	1.06	.02
Psoriasis duration (yr)	1.05	.09	NA	–
Age of onset (yr)	1.046	.13	NA	–
BMI (kg/m^2^)	1.091	.12	NA	–
UGAP (dB/cm/MHz)	212.055	.04	434.17	.03

BMI = body mass index, UGAP = ultrasound-guided attenuation parameter.

### 3.4. Analysis of factors influencing UGAP values

**Figure 2.**
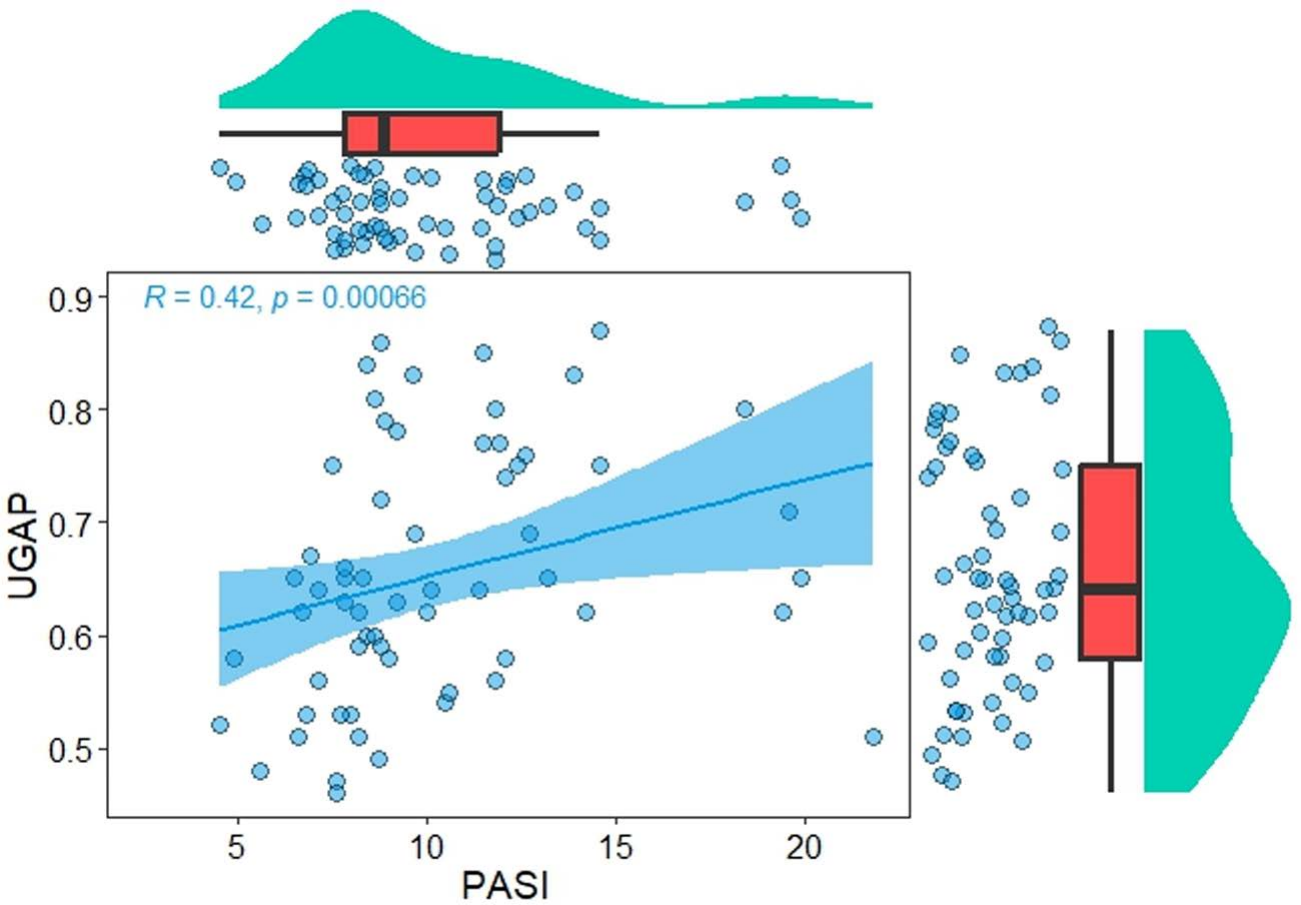
Relationship between UGAP and PASI in patients with psoriasis. The correlation efficient (R) and *P*-value were calculated using Spearman rank correlation coefficient. PASI = psoriasis area and severity index, UGAP = ultrasound-guided attenuation parameter.

Spearman correlation analysis showed that in the normal population, UGAP values were correlated with weight, BMI, GGT, and ALT (*P* < .05). Conversely, in the psoriasis population, UGAP values were correlated with body weight, BMI, and PASI, with a correlation coefficient of 0.424 (Fig. 2). No significant correlation existed between the UGAP values and other factors.

## 4. Discussion

Psoriasis is a genetically and environmentally induced immune-mediated, chronic, relapsing, inflammatory, systemic disease. It has a systemic inflammatory state that induces adipose tissue inflammation, insulin resistance, and consequently multiple systemic disorders, including psoriatic arthritis, cardiovascular disease, obesity, and MetS.^[13–16]^ MAFLD is currently the most common cause of chronic fatty liver worldwide. It is characterized by an alcoholic fatty liver-like presentation in patients who do not consume excessive amounts of alcohol. Obesity and MetS are known risk factors for MAFLD.^[17,18]^ MAFLD and psoriasis share the specific pro-inflammatory mediators interleukin-6 (IL-6), TNF, and C-reactive protein (CRP),^[8]^ the production of pro-inflammatory adipocytokines can also contribute to the accumulation of triglycerides, leading to fatty liver disease.^[19]^ Thus, patients with psoriasis may have a linked pathogenesis to MAFLD.

Liver biopsy is not the first choice for clinical diagnosis because of its invasiveness and poor reproducibility. Currently, conventional Doppler ultrasound is used to assess the degree of hepatic steatosis based on the subjective judgment of the assessors. MAFLD is diagnosed with the conventional Doppler ultrasound criteria developed by the American Gastroenterological Association,^[7]^ indicating a certain correlation between psoriasis and MAFLD. Gisondi et al^[20]^ also diagnosed MAFLD based on ultrasound features, indicating that the prevalence of MAFLD is higher in psoriasis patients than in controls. However, one shortcoming is the low diagnostic accuracy of conventional ultrasound for mild steatosis when the fatty infiltration of the liver is <33%.^[21]^ The UGAP technique is a combination of ultrasound image guidance and attenuation parameter measurements, which has the advantage of good diagnostic performance for hepatic steatosis. Magnetic resonance imaging-based proton density fat fraction and liver biopsy as a reference have been reported in literature,^[22]^ also confirming the feasibility of the UGAP technique for hepatic steatosis assessment.

Our study was the first to use the UGAP technique to assess the degree of hepatic steatosis in patients with psoriasis. We found that patients with psoriasis were more likely to develop hepatic steatosis. In previous studies, the prevalence of MAFLD is significantly higher in psoriasis patients than in matched controls or healthy groups (17%–66% vs 8%–35%).^[2,3,8,20,23]^ Our study also found that the degree of hepatic steatosis was significantly higher in patients with severe psoriasis than in those with mild-to-moderate psoriasis, and that PASI scores were correlated with body weight, BMI, and UGAP values. This finding further suggested that a longer duration and severity of inflammatory involvement in psoriasis corresponded with a higher prevalence of MAFLD. The possible reason was link between the 2 diseases in terms of inflammatory factors. MAFLD is now considered to be a hepatic manifestation of the MetS, and its incidence is gradually increasing. Psoriasis, as an inflammatory, immune disease, significantly elevates the levels of inflammatory factors and cytokines, such as TNF-α and IL-6. It also significantly reduces levels of lipocalin, which can affect lipid metabolism and glucose metabolism, thereby leading to the development of metabolic diseases, including MAFLD.^[24]^ Thus, clinicians should be aware of the liver damage that psoriasis can cause during treatment. For patients with severe psoriasis, monitoring liver function, blood lipid changes, and performing ultrasonography to assess liver damage is an essential part of clinical diagnosis and treatment. Meanwhile, for patients with liver involvement, drugs that aggravate liver injury should be avoided to optimize the treatment strategy, better select the treatment population, and realize precise medical treatment.

MAFLD is now the leading cause of increased GGT and ALT.^[25]^ In our study, GGT was significantly higher in patients with psoriasis than in the nonpsoriasis population, whereas no significant difference in GGT existed between patients with severe psoriasis and those with mild-to-moderate psoriasis. We can assume that the development of psoriasis led to a certain extent to liver steatosis, further leading to elevated GGT. However, GGT did not necessarily increase synchronously with the progression of psoriasis. Our results also showed no significant difference in the liver function indices AST and ALT, as well as the lipid parameters CHO, TG, HDL-C, and LDL-C between patients with severe psoriasis and those with mild-to-moderate psoriasis. This finding may be due to the fact that hepatic steatosis caused by psoriasis has an insidious onset with slowly progressing damage. It does not lead to abnormalities of blood lipids or significantly impaired liver function in its early stages. Conversely, the sensitivity of UGAP for the diagnosis of mild fatty liver was better than that of conventional ultrasound and laboratory tests. This finding suggested that liver function monitoring in patients with psoriasis should not be based on liver function tests alone. As an emerging noninvasive way to assess hepatic steatosis, UGAP has excellent diagnostic performance and can be extensively used as an effective technique to diagnose fatty liver in the future.

The dysfunction of adipose tissue and the low-level inflammatory response in patients with psoriasis lead to an increase in the synthesis of TG and a decrease in their oxidation, utilization and transportation, resulting in aggravated fatty liver deposition.^[15]^ Subsequently, damaged liver cells release reactive oxygen species, CRP, IL-6, and other pro-inflammatory cytokines that further drive low-grade inflammation, which can exacerbate psoriasis.^[3]^ Our study found that age and UGAP values were independent influences on PASI scores in patients with psoriasis, and an Italian study has also found MAFLD to be an independent predictor of PASI scores.^[3]^ We found that weight and BMI were positively correlated with UGAP values in normal and psoriasis patients. Therefore, we believe that UGAP value can be a reliable assessment result to prompt clinicians’ timely intervention in the progression of psoriasis and its complications. Furthermore, Severe liver steatosis can also participate in the pathogenesis of MetS and diabetes mellitus. Some patients may also have metabolic dysfunction-associated steatohepatitis, as well as the onset of liver fibrosis and hepatocellular carcinoma.^[26,27]^ Hepatotoxicity is one of the important adverse reactions of the traditional drug methotrexate for treating psoriasis. The rate of liver fibrosis and cirrhosis in patients with cumulative dose >5.0 g of methotrexate is as high as 25%. Moreover, obesity, hepatitis and diabetes can increase the risk of hepatotoxicity.^[28]^ For patients with psoriasis having a high UGAP value, the progression of liver steatosis should be controlled as early as possible. For those with abnormal transaminase levels or who have already experienced liver toxicity reactions, it is recommended to switch to safer systemic drugs as soon as possible.

However, this study had some limitations. Firstly, this study lacks liver biopsy as the gold standard for diagnosing MAFLD, because liver biopsy is an invasive procedure and is rarely used for diagnosing MAFLD in clinical practice. Secondly, there is no expert consensus on the severity grade of hepatic steatosis as reflected by UGAP. Therefore, we did not investigate whether differences would exist in PASI scores and clinical presentation among patients with different degrees of MAFLD in the psoriasis population. Such an investigation can be conducted in the future.

## 5. Conclusion

Our research findings demonstrate a bidirectional association between hepatic steatosis and psoriasis. Psoriatic patients exhibit significantly higher predisposition to hepatic steatosis compared to nonpsoriatic populations, with the severity of hepatic lipid accumulation demonstrating positive correlation with psoriasis progression. Notably, age and UGAP values emerge as independent predictive factors for psoriasis advancement. Patients exhibiting more pronounced hepatic steatosis demonstrate elevated risks of psoriasis exacerbation. In clinical practice, particular caution should be exercised when selecting antipsoriatic therapeutics for patients with severe hepatic steatosis to prevent iatrogenic liver injury. We strongly recommend implementing regular hepatic function surveillance and systematic monitoring protocols for this patient population. The UGAP measurement shows promising clinical utility as a reliable quantitative ultrasound modality for hepatic steatosis assessment, potentially establishing novel standardized parameters for comprehensive clinical evaluation.

## Author contributions

**Conceptualization:** Zhi Yang.

**Data curation:** Yuting Jiao, Shengwei Zhou, Yaqi Fang, Huantong Duan.

**Writing – original draft:** Xiaonan Wang.
